# Promoting Coordinated Development of Community-Based Information Standards for Modeling in Biology: The COMBINE Initiative

**DOI:** 10.3389/fbioe.2015.00019

**Published:** 2015-02-24

**Authors:** Michael Hucka, David P. Nickerson, Gary D. Bader, Frank T. Bergmann, Jonathan Cooper, Emek Demir, Alan Garny, Martin Golebiewski, Chris J. Myers, Falk Schreiber, Dagmar Waltemath, Nicolas Le Novère

**Affiliations:** ^1^Computing and Mathematical Sciences, California Institute of Technology, Pasadena, CA, USA; ^2^Auckland Bioengineering Institute, University of Auckland, Auckland, New Zealand; ^3^The Donnelly Centre, University of Toronto, Toronto, ON, Canada; ^4^BioQuant/Centre for Organismal Studies (COS), University of Heidelberg, Heidelberg, Germany; ^5^Department of Computer Science, University of Oxford, Oxford, UK; ^6^Computational Biology, Memorial Sloan-Kettering Cancer Center, New York, NY, USA; ^7^Scientific Databases and Visualization, Heidelberg Institute for Theoretical Studies (HITS), Heidelberg, Germany; ^8^Department of Electrical and Computer Engineering, University of Utah, Salt Lake City, UT, USA; ^9^Faculty of Information Technology, Monash University, Melbourne, VIC, Australia; ^10^Institute of Computer Science, University Halle-Wittenberg, Halle, Germany; ^11^Department of Systems Biology and Bioinformatics, University of Rostock, Rostock, Germany; ^12^Babraham Institute, Cambridge, UK; ^13^European Molecular Biology Laboratory-European Bioinformatics Institute, Cambridge, UK

**Keywords:** standardization, data sharing, reproducible science, computational modeling, biology, file formats

## Abstract

The Computational Modeling in Biology Network (COMBINE) is a consortium of groups involved in the development of open community standards and formats used in computational modeling in biology. COMBINE’s aim is to act as a coordinator, facilitator, and resource for different standardization efforts whose domains of use cover related areas of the computational biology space. In this perspective article, we summarize COMBINE, its general organization, and the community standards and other efforts involved in it. Our goals are to help guide readers toward standards that may be suitable for their research activities, as well as to direct interested readers to relevant communities where they can best expect to receive assistance in how to develop interoperable computational models.

## Introduction

Interpreting the staggering amount of biological data available today is a daunting challenge. In response, many biologists have turned to computational methods to organize their data in a coherent fashion, synthesize formal descriptions of their theories, analyze their hypotheses mathematically, and use the results to develop testable predictions. A wealth of resources is available to support these activities. For example, a large number of electronic data sources exist with content ranging from experimentally derived properties of molecular entities and biochemical reactions, through molecular interaction pathways, up to fully specified computational simulations. Many software systems also exist for supporting all parts of the spectrum of relevant activities from data processing to advanced simulation, analysis, and visualization.

The availability of appropriate data formats and process descriptions is an essential enabler for reproducible science. Researchers must be able to build on each other’s work to develop a deeper understanding of biological phenomena, but this task is greatly impeded if they do not use common languages to describe their work. In the past two decades, this has led to the development of several formats and minimum information guidelines to facilitate the exchange of data and models. However, the existence of uncoordinated standards risks creating silos that induce new interoperability problems. In an effort to prevent this, a number of community standardization efforts created COMBINE, the *COmputational Modeling in BIology NEtwork*.

## Mission and Organization of COMBINE

The Computational Modeling in Biology Network was formed in 2009 following the observation that many efforts shared similar goals and sometimes even involved the same individuals, yet organized separate workshops year after year and rarely attempted to coordinate activities or reuse common resources. The leaders of the efforts realized that many benefits could accrue from co-locating meetings, as well as cooperating on the creation of common infrastructure, common operating procedures, and potentially, a common voice to seek additional financial support.

The primary aim of COMBINE is to act as a coordinator, facilitator, and resource for different community-based standardization efforts in the area of computational biology. In this respect, it shares similar goals as other consortia in biology, such as the Genomics Standards Consortium (Sterk et al., 2010), but with a greater emphasis on standards applicable to modeling of biological phenomena. COMBINE helps foster greater interaction and awareness of the activities in different standards’ development, which encourages the federated projects to develop standards that are more likely to be interoperable and less likely to overlap substantially than if the efforts proceeded separately. COMBINE offers an infrastructure for specification documents, announcement lists, and more, as discussed below. Building on the experience of mature standards, which already have stable specifications, software support, user bases, and community governance, COMBINE also supports emerging efforts aimed at filling gaps or addressing new needs in the overall interoperability landscape. However, COMBINE does *not* dictate what individual standardization efforts should do; ultimately, the implementation of standards development processes is up to the leaders and members of the communities involved in the individual efforts.

## The Major Standards in COMBINE Today

Table 1 summarizes the standardization efforts in COMBINE today. The following sections describe the six core COMBINE standards in greater detail.

**Table 1:** **Standardization efforts in COMBINE today**.

Category	Name	COMBINE web page or other reference
COMBINE standards	BioPAX (Biological Pathways Exchange)	co.mbine.org/standards/biopax
	CellML	co.mbine.org/standards/cellml
	SBGN (Systems Biology Graphical Notation)	co.mbine.org/standards/sbgn
	SBML (Systems Biology Markup Language)	co.mbine.org/standards/sbml
	SBOL (Synthetic Biology Open Language)	co.mbine.org/standards/sbol
	SED-ML (Simulation Experiment Description Markup Language)	co.mbine.org/standards/sed-ml
Associated standardization efforts	COMBINE archive	co.mbine.org/standards/omex
	BioModels.net Qualifiers	co.mbine.org/standards/qualifiers
	Identifiers.org URIs	co.mbine.org/standards/miriam
	KiSAO (Kinetic Simulation Algorithm Ontology)	co.mbine.org/standards/kisao
	MIASE (Minimum Information About a Simulation Experiment)	co.mbine.org/standards/miase
	MIRIAM (Minimal Information Required In the Annotation of Models)	co.mbine.org/standards/miriam
	SBO (Systems Biology Ontology)	co.mbine.org/standards/sbo
Related standardization efforts	BioSharing	Sansone et al. (2012)
	CNO (Computational Neuroscience Ontology)	Le Franc et al. (2012)
	FieldML (Field Markup Language)	Britten et al. (2013)
	GPML (GenMAPP Pathway Markup Language)	van Iersel et al. (2008)
	MAMO (Mathematical Modeling Ontology)	Zhukova et al. (2014)
	NeuroML	Gleeson et al. (2010)
	NuML (Numerical Markup Language)	Dada et al. (2010)
	PSI-MI (Proteomics Standards Initiative)	Hermjakob et al. (2004a)
	SpineML (Spiking Neural Markup Language)	Richmond et al. (2014)
	TEDDY (TErminology for the Description of mDYnamics)	Courtot et al. (2011)

### Biological Pathway Exchange

The *Biological Pathway Exchange* (BioPAX)^1^ is an RDF/XML (Lassila and Swick, 1999) based format that focuses on exchanging and integrating large biological process maps (Demir et al., 2010). There are currently more than 500 pathway databases that curate this information from the literature and other sources (Bader et al., 2006). Many of these groups originally developed their own representations, conventions, and controlled vocabularies, making it extremely difficult to combine and use pathway information from multiple sources. BioPAX was created by a community of pathway database groups, tool developers, and scientists to facilitate data exchange and integration.

Biological Pathway Exchange Level 3, released in 2010, can represent metabolic and signaling pathways, gene regulation networks, and molecular complexes as well as molecular and genetic interactions. BioPAX can capture detailed information about these processes including post-translational modifications and subcellular location of participants. BioPAX also stores information about the scientific support for pathway data including references to articles, experimental evidence, and confidence. Whenever possible, BioPAX uses existing controlled vocabularies for annotating entities, such as Gene Ontology (The Gene Ontology Consortium, 2000) for cellular locations, the PSI-MOD (Montecchi-Palazzi et al., 2008) controlled vocabulary for describing post-translational modification and the PSI-MI (Hermjakob et al., 2004b) controlled vocabularies for experimental evidence.

Biological Pathway Exchange-formatted pathway data can be used to explore pathways and interactions, to analyze high-throughput omics data in the context of pathways, and as a blueprint for the development of models that can be simulated. BioPAX can be visualized best in SBGN-PD and can be converted to SBML for quantitative analysis.

### CellML

CellML^2^ is an XML-based format that provides a modular framework for the encoding of mathematical models (Cuellar et al., 2003). The primary focus of CellML is the encoding of models consisting of differential algebraic equations. The mathematical model, expressed using MathML (Ausbrooks et al., 2003), is considered to be the primary data and biological context is provided by annotating the variables and equations with metadata using RDF (Lassila and Swick, 1999). All numerical values and variables used in a CellML document are required to unambiguously define their physical units.

At its core, CellML defines lightweight XML constructs that group mathematical relationships within modules. The variables used in the mathematics are defined within each module and connections between variables in different modules can be specified. Due to the requirement for physical units, numerical quantities can vary between modules and software is expected to convert units automatically. CellML models are able to define hierarchies of modules to enable mathematical abstraction, and hierarchical modules are able to be imported from external CellML models. This enables the reuse of models, or parts of models, in a generic manner.

### Systems Biology Graphical Notation

The *Systems Biology Graphical Notation* (SBGN)^3^ standardizes the visual notation used to depict biological networks and processes (Le Novère et al., 2009). The use of a standard visual notation is vital to ensure that diagrams are unambiguous and consistent; it also promotes the development of better software tools for authoring diagrams.

Systems biology graphical notation defines three languages: *Process Description*, *Activity Flow*, and *Entity Relationship*. PD can describe each process in a network in great detail (e.g., biochemical reaction, binding/unbinding of proteins, and the like) and is useful to represent chemical kinetics models. However, some biological phenomena entail a combinatorial explosion of possible interrelated states, making them extremely difficult to depict at this level of detail. ER maps are more suitable to these cases because they abstract away the notion of time and focus on depicting only the relationships between elements, independent of each other. ER is useful to represent rule-based models. Finally, AF maps focus on the influences between elements rather than the actual processes, and are useful for representing qualitative models.

Systems Biology Graphical Notation can be used to visualize data and models in BioPAX, SBML, and CellML formats; work is currently underway to connect SBGN and SBOL as well. The SBGN website provides an overview of many software systems supporting SBGN, and a large collection of SBGN diagrams can be found at the Path2Models project website (Büchel et al., 2013).

### Systems Biology Markup Language

The *Systems Biology Markup Language* (SBML)^4^ is a machine-readable representation format for computational models in systems biology (Hucka et al., 2003). In SBML, models are decomposed into explicitly labeled constituent elements (e.g., substances involved in processes, compartments where they are located); models are *not* cast directly into a specific form such as differential equations. SBML also neither encode what is *done* with a model nor the results of doing something with it – these are aspects addressed by other COMBINE standards such as SED-ML. This abstract approach makes it possible for a software tool to translate the SBML form of a model into whatever internal form the tool actually uses, whether that be differential equations, stochastic systems, or some other framework; it also makes it possible to use the same model for other types of analyses besides dynamical simulation. Support for SBML has been implemented in over 260 software systems (both open-source and commercial) to date.

The evolution of SBML proceeds in stages in which each “Level” is an attempt to achieve a consistent language at a certain level of complexity. SBML Level 3 is modular, with the core usable in its own right and *Level 3 packages* being additional “layers” that add features to the core. By itself, core SBML Level 3 is well suited to representing such things as classical metabolic models and cell signaling models, involving well-mixed substances and spatially homogeneous compartments where they are located. Other model types can also be expressed using SBML’s core constructs, but SBML Level 3 packages add more natural support for such types as qualitative models (e.g., Boolean network models), constraint-based models, rule-based models, and spatially inhomogeneous processes. The list of SBML Level 3 package activities (over a dozen today) can be found on the SBML website.

### Synthetic Biology Open Language

The *Synthetic Biology Open Language* (SBOL)^5^ is a proposed standard for describing genetic parts and engineered designs in synthetic biology (Galdzicki et al., 2014). SBOL consists of collections of annotated *DNA component* sequences. For example, a DNA component may be a segment of DNA that has a particular function such as a *promoter*, *open reading frame* (i.e., gene), *ribosome binding site*, *terminator*, etc. The type of a component is indicated using a type from the *sequence ontology* (Eilbeck et al., 2005). A component can also be a sequence that is composed of other components hierarchically. Each annotation indicates the start and end point of the annotation within the sequence, and also the strand on which it is located in the case of DNA components. The order of annotations can also be given using the SBOL *precedes* relation when the sequence is not yet known.

*Synthetic Biology Open Language* is the youngest standard in COMBINE, but it is rapidly gaining new followers beyond the 40 organizations currently in the SBOL community. Current major directions for evolution include extending SBOL beyond its current support for only structural information about DNA components. For example, extensions now under development include modules and their connections, with modules having associated models defined in SBML or CellML format and interactions defined by terms drawn from the Systems Biology Ontology (SBO).

### Simulation Experiment Description Markup Language

The *Simulation Experiment Description Markup Language* (SED-ML)^6^ is an XML format to encode descriptions of simulation protocols (Waltemath et al., 2011). These standardized descriptions ensure that virtual experiments, when applied to a computational model, reproduce a given result. Similarly to SBML, SED-ML evolves in *Levels* and *Versions*.

Simulation Experiment Description Markup Language comprises a reference to the models being used in the simulation; descriptions of modifications applied to the model before simulation; descriptions of the simulation steps, including the configuration of the software tool or numerical algorithm; descriptions of the post-processing of result data after simulation; and specifications of the results to be provided to the users. Simulation algorithms are characterized with terms from the Kinetic Simulation Algorithm Ontology KiSAO (Courtot et al., 2011). Modifications before and after simulation are described using MathML (Ausbrooks et al., 2003), the web standard for describing mathematical expressions in XML form.

Simulation Experiment Description Markup Language files can be linked to model descriptions in other formats, notably SBML or CellML, to ensure reproducibility of experiments presented in scientific publications. The links can, for example, be instantiated on the storage layer (Henkel et al., 2015), via the provision of files in a COMBINE archive (Bergmann et al., 2014), or through provision via public model repositories such as BioModels Database (Li et al., 2010) or the Physiome Repository (Yu et al., 2011).

## Activities Performed by COMBINE

How does COMBINE fulfill its aim of promoting greater awareness, discussion, and collaboration in the development of information standards for computational biology applications? The following are the consortium’s main activities:

- *Organize meetings*: COMBINE organizes open meetings where interested people can gather for face-to-face discussions and work on standards. The primary meetings are the annual COMBINE Forum and the annual HARMONY (HAckathon on Resources for MOdeliNg in biologY) workshop, held approximately 6 months apart. The joint meetings help the different standardization efforts work together; they also make financial sense by reducing the overall number of meetings, travel, and money spent on hosting meetings. (However, COMBINE does not currently have any funding of its own, and the meetings must be organized by groups that volunteer to host them.) The leaders of the various standards also endeavor to write meeting reports that summarize the outcomes of the meetings (e.g., Le Novère et al., 2011; Waltemath et al., 2014).
- *Help coordinate standards development*: Thanks in large part to the meetings that COMBINE organizes, the discussion forums it provides, and the involvement of many of the same people in multiple standardization efforts, COMBINE helps coordinate the activities of the different efforts. This reduces duplication of effort, user confusion, and non-interoperability among the efforts.
- *Identify missing standards and initiate efforts to develop them*: COMBINE’s meta-community is in an ideal position to identify what is missing from the current constellation of standards in computational systems biology. This has already yielded benefits: we have recently developed the COMBINE archive, a format that fills the need for a simple, consistent way of bundling multiple files related to a modeling project^7^ (Bergmann et al., 2014); and we have also begun to identify missing minimal requirements for common annotations across the spectrum of data used in biological modeling, such as parameter identifiability (tentatively called the Minimal Information for Model Inference and Parametrization – MIMIP) and mathematical classification (the Mathematical Modeling Ontology – MAMO).
- *Provide a specification infrastructure*: COMBINE provides a consistent framework for cataloging the definitions of COMBINE standards. This framework includes a consistent, hierarchical identifier scheme for identifying standard specifications; a URI scheme for locating specifications and standards using Identifiers.org to provide permanent, resolvable URIs for standards (Juty et al., 2012) and a web page structure for the description of each standard.
- *Develop common procedures*: Many standardization efforts are started by academics, which have little experience with community organization. Effective organization is something that takes time and experience to learn. In COMBINE, we are documenting our experiences and collecting them into a collection of examples, recommendations, and best practices^8^. We hope to provide would-be standards developers with a set of off-the-shelf “standard operating procedures” for different situations and goals.
- *Organize tutorials*: Educating biologists about available standards and compatible software tools is another important activity pursued by COMBINE. We organize tutorials at the primary COMBINE meetings as well as at international conferences, in particular the annual *International Conference on Systems Biology* (ICSB).
- *Maintain collective online forums/groups*: COMBINE maintains mailing lists and online discussion forums^9^. A discussion list cover the topic of general interest for all COMBINE members, while dedicated lists cover specific issues such as the COMBINE archive, metadata, etc. General announces are done via social media (e.g., Twitter feed *@combine_coord*).

An additional activity that we hope to undertake soon is fund-raising. This will require COMBINE to become a legal entity that can accept funding. Once this is in place, we hope to be able to fund the meetings and online infrastructure, and perhaps also seek funding for further standards development.

## Conclusion

Computational modeling has been used to help elucidate biological phenomena for decades, with some work worthy of Nobel prizes (Hodgkin and Huxley, 1952). In this data-driven age of biology, modeling has become more relevant than ever as a means of drawing insight from data. Maximum reusability of models is paramount, in all situations from publications to public databases. However, reusability is practically impossible without agreement about the formats used to store and exchange the models. Without standards, the diversity of software tools available today would make it difficult for researchers to use multiple software tools in their work. Different software tools today are implemented in different programing languages, run on different operating systems, express models using different mathematical frameworks, provide different analysis methods, present different user interfaces, and support different file and data formats. Exporting a model from one tool and importing it in another is difficult or impossible unless both tools understand the same format. Better coordination of formats used by software tools thus removes obstacles to research. In so doing, it enhances opportunities in computational modeling, a challenging activity that requires careful formulation of questions, selection of appropriate methods, and a certain “computational” way of thinking (Wing, 2006; Rubinstein and Chor, 2014).

The standardization efforts involved in COMBINE strive to facilitate greater reusability by developing tool-independent, open standards for a range of needs, including models, metadata, ontologies, and protocols. The COMBINE umbrella also facilitates interactions between new standards initiatives and established standardization efforts, enabling the new initiatives to take advantage of the existing experiences and expertise. Finally, COMBINE performs an advocacy role by promoting the adoption of standards-based methods and software tools via tutorials, workshops, and focused sessions at international conferences. Members of COMBINE also work with journal editors and publishers to promote the adoption of standards-based guidelines for the publication of modeling studies.

All of the community standards in COMBINE have open and freely available specifications, and have no licensing or other restrictions. The COMBINE consortium has begun to liaise with official national standardization bodies (e.g., the German DIN), as well as the International Organization for Standardization (ISO) with the aim to promote and distribute the COMBINE standards to a broader user community that includes industries and governmental organizations. As these user communities often rely on certification of standards by official standardization bodies, COMBINE is seeking to find ways of getting official recognition of *de facto* standards that are already accepted and used in academic research. If successful, such efforts will expand the reach of COMBINE standards and widen their user community beyond the academic world; it may also help open new avenues for obtaining long-term support for the standards. Throughout this undertaking, we are committed to maintaining the openness of COMBINE standards, to ensure that anyone may freely use them without restrictions due to licensing or other intellectual property encumbrances.

The ultimate goal of improved interoperability between data and software resources is to improve scientists’ ability to reuse data and models from a range of sources, both public and private. This kind of reuse in turn significantly enhances scientists’ ability to repeat or reproduce previous studies, thereby aiding verification and validation. COMBINE is an open organization and we invite everyone who is interested in pursuing these goals to join the organization, get involved, and help improve the community standards for modeling in biology.

## Author Contributions

All authors contributed to the conception and writing of this manuscript and have reviewed and approved the submitted version.

## Conflict of Interest Statement

The authors declare that the research was conducted in the absence of any commercial or financial relationships that could be construed as a potential conflict of interest.

## Footnotes

^1^http://biopax.org

^2^http://cellml.org

^3^http://sbgn.org

^4^http://sbml.org

^5^http://sbolstandard.org

^6^http://sed-ml.org

^7^http://co.mbine.org/standards/omex

^8^http://co.mbine.org/Documents/criteria

^9^http://co.mbine.org/comm
